# Identification and Characteristics of microRNAs from Army Worm, *Spodoptera frugiperda* Cell Line Sf21

**DOI:** 10.1371/journal.pone.0116988

**Published:** 2015-02-18

**Authors:** Pavan Kumar Kakumani, Mahendran Chinnappan, Ashok K. Singh, Pawan Malhotra, Sunil K. Mukherjee, Raj K. Bhatnagar

**Affiliations:** 1 Insect Resistance Group, International Centre for Genetic Engineering and Biotechnology, Aruna Asaf Ali Marg, New Delhi, 110067, India; 2 Department of Zoology, University of Delhi, Cavalry Lane, New Delhi, 110007, India; 3 Malaria Group, International Centre for Genetic Engineering and Biotechnology, Aruna Asaf Ali Marg, New Delhi, 110067, India; 4 Plant Molecular Biology Group, International Centre for Genetic Engineering and Biotechnology, Aruna Asaf Ali Marg, New Delhi, 110067, India; Ecole des Mines d'Alès, FRANCE

## Abstract

microRNAs play important regulatory role in all intrinsic cellular functions. Amongst lepidopteran insects, miRNAs from only *Bombyx mori* have been studied extensively with a little focus on *Spodoptera sp*. In the present study, we identified a total of 226 miRNAs from *Spodoptera frugiperda* cell line Sf21. Of the total, 116 miRNAs were well conserved within other insects, like B. *mori, Drosophila melanogaster* and *Tribolium castenum* while the remaining 110 miRNAs were identified as novel based on comparative analysis with the insect miRNA data set. Landscape distribution analysis based on Sf21 genome assembly revealed clustering of few novel miRNAs. A total of 5 miRNA clusters were identified and the largest one encodes 5 miRNA genes. In addition, 12 miRNAs were validated using northern blot analysis and putative functional role assignment for 6 S*f* miRNAs was investigated by examining their relative abundance at different developmental stages of *Spodoptera litura* and body parts of 6^th^ instar larvae. Further, we identified a total of 809 potential target genes with GO terms for selected miRNAs, involved in different metabolic and signalling pathways of the insect. The newly identified miRNAs greatly enrich the repertoire of insect miRNAs and analysis of expression profiles reveal their involvement at various steps of biochemical pathways of the army worm.

## Introduction

miRNAs (miRs) are a class of small, non-coding RNAs, from 19–24 nt in length, that are produced by all animals and plants to regulate gene expression. Since the discovery of first miRs, lin-4 and let-7 from *Caenoharbditis elegans* [1, 2, 3] hundreds of miRs have been identified to date [4]. More than 30,000 miRs have been identified from different species, such as *Spodoptera litura* [5], *Bombyx mori, C. elegans, Arabidopsis thaliana* and *Homo sapiens*, by either computational or experimental method and deposited in the miRBase [miRBase v19]. The miRs play important roles in many physiological processes, such as growth, development, metabolism, behaviour and apoptosis through post transcriptional regulation by binding to complementary regions of messenger RNA in the 3’ untranslated region (UTR), 5’ UTR and the coding region [6, 7, 8, 9, 10, 11, 12, 13, 14]. In humans, it has been estimated that, approximately 35–70% of different mRNA transcripts are regulated by miRs, with each miR potentially regulating hundreds of transcripts [15].

The miR genes are expressed mainly by RNA polymerase II in the nucleus forming the primary miR (pri-miR) [16]. A pri-miR is cleaved by Drosha to a 60–80 nt precursor miR (pre-miR), which is then exported to the cytoplasm and cleaved by Dicer-1, generating an approximately 22 nt double-stranded miR (miR:miR* duplex). Usually, miR* (miRStar) is degraded, and the miR binds to mRNA for processing by RNA-induced silencing complex (RISC) [17]. Mature miRs are used as guide RNAs to direct RISC to complementary regions of mRNAs, resulting in the inhibition of translation and or target mRNA cleavage. In animals, complementarity of miRs with their target sequences is partial [18] unlike in plants. Perfect pairing between a target and nucleotides 2–8 of the miR (seed region) usually plays a significant role in target recognition [15].

So far, the research of miR mainly focuses on mammals (such as *H. sapiens* and *Mus musculus*) and eudicotyledons (Such as *Medicago truncatula* and *A. thaliana*). Insect miR identification is far behind nematodes, mammals and plants. A total of about 2300 insect miRs have been identified from 22 insect species so far, including *Drosophila melanogster, Anopheles gambie, Apis melifera, B. mori* and *Drosophila pseudoobscura* and deposited in miRBase. Most of these insect miRs were identified by computational method and have not been experimentally validated. A few recent reports have highlighted the importance of miRs in developmental stages, metabolism and in response to viral infection from polyphagous *Spodoptera sp* [5, 19]. With the identification of new miRs in a number of organisms, evolutionary sequence conservation has become a hallmark of miR biology, especially in insects [20, 21, 22, 23].

The identification, sequence analysis of miRs from diverse and closely related organisms has revealed several important features. It is clear that, certain miRs are conserved across insect, mammals while within insects; a close phylogenetic relationship is distinctly discernible. Nevertheless, species specific miRs also exist, reflecting system-specific characteristics. The occurrence of conserved miRs in different tissues and at various developmental stages in closely related species is reminiscent of conservation of biological functional regulatory network. At the same time, the genus specific miRs suggest evolutionary divergences that impart uniqueness to the organism.

In the present study, we profiled of miR population from lepidopteran cell line, Sf21 (Life Technologies, USA), derived from the ovary of *S. frugiperda* (*Sf*). Using deep sequencing technology and computational approaches, we identified conserved as well novel miRs from *S. frugiperda* based on draft genome of Sf21 cells as a reference [24]. The miRs were investigated for their homology with other insects and studied their characteristics. In addition, we examined expression pattern of a few selected *Sf* miRs in different developmental stages as well body parts of *S. litura* larvae. Further analysis identified potential target genes for the selected miRs, including the KEGG pathways associated with them. Studies in continuation to these observations would facilitate understanding regulatory role of miRNAs in the biology of *S. frugiperda*.

## Materials and Methods

### Cell lines and Insects

Sf21 cell line was maintained in Baculo Gold TNM-FH medium with 10% FBS at 27°C. The strains of *S. litura* were maintained at Department of Zoology, University of Delhi, New Delhi and were reared on an artificial diet under 28°C at a photo period of Light: Dark 14:10 hours.

### Preparation and sequencing of small RNA library

Total RNA was isolated from Sf21 cells using Trizol as per manufacturer’s instructions (Life Technologies, USA). The small RNA library was prepared from the total RNA using TrueSeq Small RNA preparation guide (Illumina Inc., USA). Briefly, cDNA was prepared from the total RNA using adapter primers and was resolved on 6% PAGE gel. The gel fragment corresponding to miR population was excised and the library was recovered. Subsequently, the cDNA was analyzed on Agilent Technologies 2100 Bioanalyzer and run on Illumina sequencing platform at University of Delhi South Campus, New Delhi, India.

### Analysis of small RNA sequencing data

Sequencing of small RNA library using Illumina GAIIx platform generated small RNA reads of 33 bp in length. After initial quality check, the raw reads were taken for adapter trimming and filtering of bad quality data (reads containing poly N and poly A). After clipping adapters, the read length varied from 5 to 33 bps. For downstream analysis, only the reads having length between 18 to 33 bps were considered.

### Identification of microRNAs

The high quality reads with read count more than 10 were used for investigating the small RNA population. The small RNA reads were converted into unique read tags and compared against the Noncode database using BLASTN. Those read tags having 100% query coverage with maximum 2 mismatches with the known data were then distributed into different small RNA families.

To identify the known miRs, the mature sequences from the species *A. gambiae, Aedes aegypti, Culex sp, D. melanogaster, B. mori, A. mellifera, Acyrthosiphon pisum, Tribolium castaneum* and *C. elegans* were downloaded from miRBase v19. Using miRCat, the small RNA reads were mapped against the known set of mature sequences and those reads satisfying the set criteria were separated and named based on their subject annotation. Further, those annotated reads were mapped with minimum 90% query coverage and 90% identity having no gaps to the draft genome assembly of *S. frugiperda* [24] to extract pre miR sequences. The average length of precursor miR was 135 bp and the structure validation was done using MiPred to classify between real and pseudo precursors using random forest prediction model.

The small RNA reads not mapped to Noncode and miRBase were used to identify novel miRs. The small RNA reads were mapped against the draft genome assembly [24] of *S. frugiperda* using Bowtie. Once the mature sequences were mapped, the miR loci were identified using miRCat [25] and the small RNA reads with higher read counts were considered as the putative. Flanking sequences surrounding the selected miR were extracted with varying window lengths and folded into secondary structures using RNA fold. The secondary structures were further validated using the criteria for novel miR identification [25].

### Northern blotting

Total low-molecular-weight RNA was isolated from cultured Sf21 cells using mirVANA miRNA isolation kit (Life Technologies, USA). 10 μg of each low-molecular-weight RNA was resolved by electrophoresis on a 15% polyacrylamide containing 8 M urea. RNA was electro blotted for 90 min at 15 V onto a Hybond-N^+^ membrane (GE Healthcare, USA) and immobilized by UV cross-linking at 1200X 100μJ. The probes were labelled with γ^32^ [P] ATP by 5’ end labelling using T4 kinase and were allowed to hybridize at RT overnight. The membrane was washed thrice in 6X SSC with 0.2% SDS and once in 6X SSC with 0.2% SDS at 42°C and then exposed to a phosphor imager screen (Amersham Biosciences, USA) overnight and scanned at 200 μm in Typhoon-9210 (Amersham Biosciences, USA).

### Expression profile of miRNAs

The miR expression levels were quantitated using TaqMan small RNA assay system from Life Technologies, USA. Briefly, total RNA from samples was isolated using Trizol. 1 to 10 ng of total RNA was used for reverse transcription using a specific RT primer with the following conditions, i.e., 16°C: 30 min, 42°C: 30 min, 85°C: 5 min. Subsequently, the cDNA was used for qRT analysis with TaqMan probes as per the manufacturer’s instructions. For all qRT based TaqMan assays, the experiments were performed 3 times. Each time, total RNA isolated from two pooled biological sets of 10 insects each was considered for PCR set up with three technical replicates. During the qRT analysis, the 2-ΔΔCT method was employed and each Ct value of the test miR was normalized to that of U6snRNA (endogenous control) of the respective sample. Once, the value for all the three experiments was recorded, a two tailed student’s t test was performed to calculate the statistical significance of the results.

### Target prediction for miRNAs

The predicted gene list from *Sf* draft genome assembly [24] was employed to extract the coding as well the UTR information for each gene. RNA hybrid was run for the selected miRs against the coding as well the UTR information for each gene and only those targets giving mi:mRNA binding energy (Minimum Free Energy (MFE)) less than -25 Kcal/mol were considered as putative target genes. The target genes were further annotated against the NCBI RefSeq invertebrate protein dataset and Gene Ontology terms were assigned based on the annotation. Further, the target genes were categorized into different pathways as per guidelines mentioned in KEGG database.

## Results and Discussion

### Analysis of small RNA library from Sf21 cells

**Fig 1:**
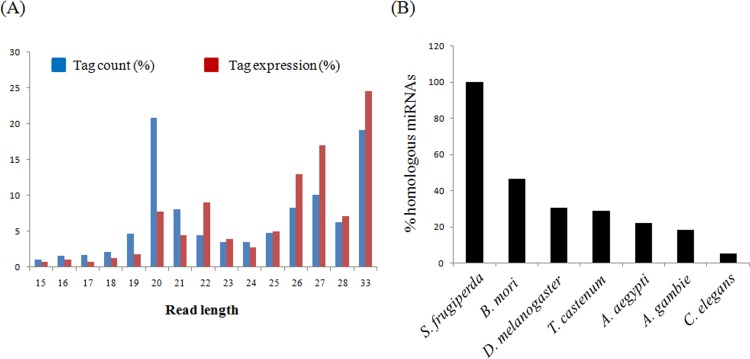
Analysis of small RNA reads and conservation of homologous miRNAs. (A) The sequence length distribution of small RNA read tags in Sf21 cells showed majority of the population at 20 nt, a typical length of mature miRNAs followed by the read lengths around 27 nt which could be putative piRNA sequences. (B) Percentage of known miRNAs from *Spodoptera frugiperda* with homologs from other insects; *B. mori, D. melanogaster, T. castenum, A. aegypti, A. gambie* and *C. elegans*.

We used next generation sequencing data to investigate small RNA population from *S. frugiperda* and identified miRs, following computational approaches. A small RNA library was prepared from total RNA of Sf21 cells and sequenced on illumina Next Gen sequencing platform. A total number of 26079325 high quality reads were obtained after adapter trimming and were distributed under unique read tags to a total of 3275664 non-redundant sequences. These sequences with an average read lengths varying between 15 and 33 were used for downstream analysis. As shown in Fig. 1A, the unique read tag of length 20 nt, which is a typical length of a mature miR, represent 20.8% of tag count and 7.7% tag expression.

Small RNA libraries from other insects as well from Sf9 cells have been reported to be rich in piRNA population [5, 19, 26, 27, 28]. Also, as shown in Fig. 1A, the small RNA library from Sf21 cells consists of read length around 27nt in significant proportions, which could be putative piRNAs. To examine this further, the small RNA reads were used for a homology based search against the Noncode database. Our analysis revealed that, the small RNA reads with better homology towards known piRNAs appear to occupy 4% of the total small RNA reads as compared to 11% being occupied by miRs. These results suggest that, Sf21 cells encode not only miRNAs but also other small RNAs such as piRNAs in significant proportions which might be involved in cell response to viral infection as reported earlier [19].

### Identification of known miRNAs from Sf21 cells

miRs are known to be conserved among different species. Here, in the present study, the trimmed high quality Sf21 small RNA reads were used to identify known miRs from *S. frugiperda*. The analysis resulted in a total of 116 known miRs and majority of homologous sequences were found in *B. mori* (Fig. 1B); in total, 65 out of 143 (46%) when compared to other insects, *D. melanogaster*; 43 out of 143 (30%), *T. castenum*; 41 out of 143 (29%). Interestingly, all the other insects *A. pisum, A. aegypti, A. gambie, A. mellifera* and *Culex sp* each share an equal proportion of homologous sequences (∼ 20%) with *S. frugiperda* while *C. elegans* share the least at 7 out of 143 (5%). Among the highly expressed; bantam, miR-184, miR-81, miR-100, miR-92, miR-2766, miR-279 are listed in the top (S1 Table) and these findings are in concurrence with recent reports [19]. These miRs also reported to be highly expressed in other insects and seem to be conserved over a large class of insect species [5, 29, 30]. These results clearly indicate that miRs can act as markers in defining evolutionary relationship between a wide range of insect species.

### Homology analysis of known miRNAs

**Fig 2:**
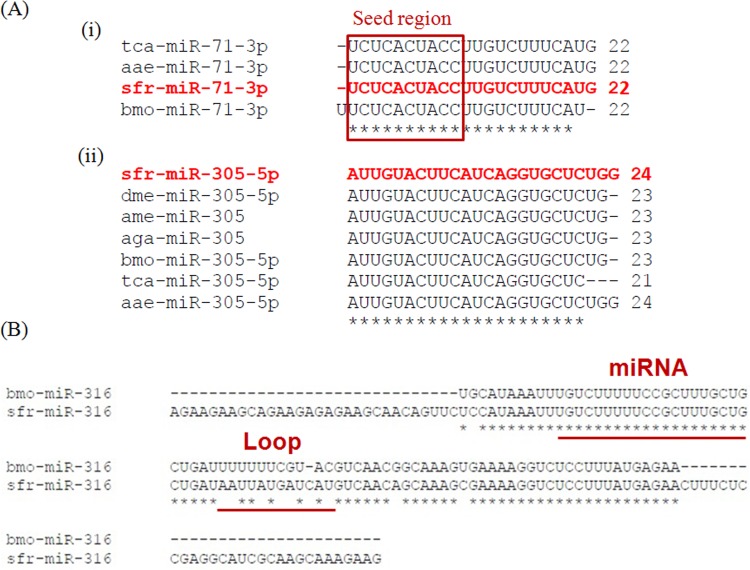
Homology analysis of *Spodoptera* miRNAs. (A) (i) Identity in the seed region of the *Spodoptera* miRNA with respect to its counterpart from other insects. (ii) Sequence conservation of the *Sf* miRNA, including the seed region over a wide range of insect species. (B) Similarity between the precursor sequences of *Sf* miRNA with its counterpart from *B. mori*. The nucleotide differences are only outside the mature region but in the stem loop sequence.

Most of the insect miRs are known to be well conserved, despite considerable diversity in the genome sequences. We carried out the homology analysis of the *Sf* miRs with known miRs from the aforementioned insect species especially from *B. mori, T. castenum* and *D. melanogaster*. The analysis revealed that, some of the known miRs (sfr-mir-305-5p, sfr-mir-307-3p) are expressed in a wide range of insect species (*A. gambie, Culex sp, A. aegypti, A. mellifera, B. mori, T. castenum, D. melanogaster*) while some (sfr-mir-71-3p) are restricted to a few (*A. aegypti, B. mori, T. castenum*). Also, it is well known that complimentary sites in targeted genes and seed sequences of miRs may be conserved in various species resulting in functional conservation of miRs [31]. As shown in the Fig. 2A, the *Sf* miRs are well conserved in sequence, and particularly, the seed region is identical to that of homologous miRs of other insects; *D. melanogaster, T. castenum, A. aegypti* and *A. gambie*. These observations implicate that, orthologous miRs may regulate the orthologs of targeted genes in related species, such as, miR-9a controlling Sensory Organ Precursors (SOPs) in *Drosophila* and *Bee* [32]. Similarly, miR-2 and miR-7 control signal transduction pathways (notch signalling) in both *Drosophila sp* and *B. mori* by targeting HLHmδ, HLHm3, HLHm5, HLHmγ, M4 and TOM [33, 34].

In addition, we compared the mature miR sequences to the draft genome assembly of *S. frugiperda* [24] with 100% identity to extract pre-miR sequences. The precursor sequences were annotated using MiPred and compared the precursor sequences of a few miRs with their counterparts from *B. mori*. Interestingly, precursor sequences of the miRs in *S. frugiperda* and *B. mori* showed a high level identity (90%, Fig. 2B). The sequence and position of the mature miR were the same. Indeed all nucleotide differences of pre-miR in *S. frugiperda* were outside the mature miR position (Fig. 2B). These findings add to the notion that, pre-miR sequences in closely related species are well conserved as reported in previous publications [30]. Also, synteny in genome at precursor level would greatly add to the study of regulatory RNAs and their functional relationship between *Spodoptera* and *Bombyx* genus.

### Identification of novel miRNAs from Sf 21 cells

To identify the novel miRs, we used the draft genome assembly of Sf21 cells as a reference [24]. The small RNA reads with read count of more than 10 were only considered for alignment with the genome scaffolds with 100% identity. The sequences satisfying the criteria were extracted to see whether they can fold into canonical pre-miR structures. The analysis identified a total of 110 novel mature miRs. Also, we observed miR* sequence for each mature miR although these were present with very low levels of read-count. The sequence and the other necessary information on the novel miRs is presented in S1 Table. Among the 122 mature miRs, 53 arise from 5’ arm while the rest arise from the 3’ arm of the stem loop structure. The length of mature miR sequences varied from 18–24nt with the major part being within 20–23nt (∼ 93%). At the 5’ end of the annotated miRs, there is a preference for a U followed by C, A and G. All of these features of the miR sequences are in good concord with known specificity of Dicer processing [35]. Here, the number of novel miRs identified is higher when compared to the findings of previous reports, pertaining to the choice of reference genome taken for analysis [19]. Also, from findings of the draft genome assembly of Sf21 cells [24], it appears that, though there are similarities between the genomes of two closely related species, *B. mori* and *S. frugiperda*, there are considerable differences in expression pattern of some of the genes identified. This would explain the reason behind the change in number and sequence of novel miRs as they regulate genes necessary for the biological relevance of *S. frugiperda*.

**Fig 3:**
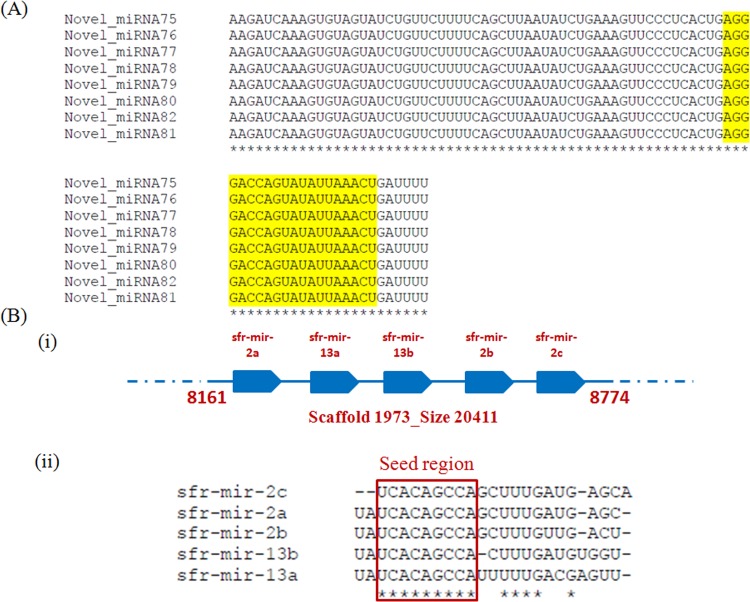
Analysis of novel miRNAs identified in the library. (A) Similarity between the precursor sequences of a miRNA family identified in the population. (B) (i) The largest miRNA cluster identified from the library using *Sf* draft genome assembly as a reference. (ii) Similarity in the nucleotide composition of the mature miRNA sequences present in the cluster, especially the seed region.

In addition, we identified 12 miR families from different genomic locations. As shown in Fig. 3A, miRs in the family (Novel_miR75-82) showed complete identity with each other. In addition, we identified miR clusters as they have been reported as a common feature in many insect species [7]. A cluster usually contains two or three pre-miR sequences although larger clusters have been identified such as six membered hsa-miR-17 cluster [36, 37, 38] or the *D. melanogaster* cluster containing eight miR genes [39]. Clustered miR genes have been shown to share a high degree of similarity in nucleotide composition but occasionally the miR sequences differ significantly [7, 40]. Examining the positions of the identified novel pre-miR sequences in the *Sf* draft genome assembly, we identified five miR clusters from different genome scaffolds with two major clusters having 5 and 3 pre-miR sequences respectively. The information on the miR clusters and the abundance of each miR in the cluster in presented in S2 Table. The cluster (sfr-mir-2a, sfr-mir-2b, sfr-mir-2c, sfr-mir-13a, sfr-mir-13b) is distributed over the region, 8161–8774 of scaffold 1973 with a size of 20411bp (Fig. 3B, i), while the other (sfr-mir-10494, sfr-mir-10463, sfr-mir-10471) span over 10636bp region of scaffold 6745. Also, as shown in Fig. 3B, ii, the seed region of the miR cluster is identical while the miR* sequence differ significantly. These findings are concurrent with previous reports that the lepidopteran miRs cluster as discovered in silkworm *B. mori* [41]. The close proximity of these novel miR genes implies that they may be transcribed and processed as a large precursor under a single regulatory element [42]. Such an operon like organization converges on the same target gene or targets a different number of genes for decreased transcription and/or translational repression [43].

### Expression of a few selected miRNAs

**Fig 4:**
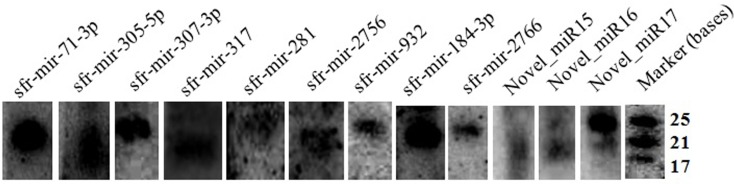
Northern validation of selected miRNAs. Both the known and novel miRNAs were checked by northern analysis for their expression in Sf21 cells using the total RNA isolated from them. All the miRNAs exhibited the expected sizes from the library.

We used northern analysis to confirm the expression of some of predicted miRs present in our sequencing data-set. A total of 12 miRs; 7 known and 5 novel miRs were checked for their expression in Sf21 cell lines. The miRs (sfr-mir-305-5p, sfr-mir-307-3p, sfr-mir-71-3p, sfr-mir-281, sfr-mir-317, sfr-mir-2756, sfr-mir-932, sfr-mir-184-3p, sfr-mir-2766, Novel_miR15, Novel_miR16, and Novel_miR17) were easily detectable in total RNA isolated from Sf21 cells (Fig. 4). In general, the detection levels of a given miR reflected the overall abundance of that miR in the sequenced library. All miRs analyzed by this method exhibited the expected sizes.

### Expression levels of the miRNAs in *S. litura* insect developmental stages

miRs are known to play crucial roles in tissue development or differentiation or maturation [5, 44]. In view of these reports, the expression pattern of some of both the known and novel miRs identified in Sf21 cells were tested in *S. litura. S. frugiperda* is restricted in distribution to Americas and Latin Americas. Its related species, *S. litura* is distributed throughout Asia and Australia. To extend the miR profile results obtained from Sf21 further, we included larvae of *S. litura* in experimental design. Analogous extension of distribution and occurrence of miRs in related insect species has been employed previously [44]. In this specific instance, miR-2a and miR-34 were identified from *Helicoverpa armigera* and *S. litura* based on *in silico* comparative analysis using data set of *B. mori*. Total RNA isolated from the larval stages was used as a template to identify the expression levels of the chosen miRs using TaqMan microRNA assays. These assays have been widely used due to their high specificity and would serve the current purpose, as the mature miR sequences from two different insects under the same genus, *S. frugiperda* and *S. litura*, would hold higher homology than the observed between two different insects under the same order, *B. mori* and *S. frugiperda*, (Fig. 3B), [19].

**Fig 5:**
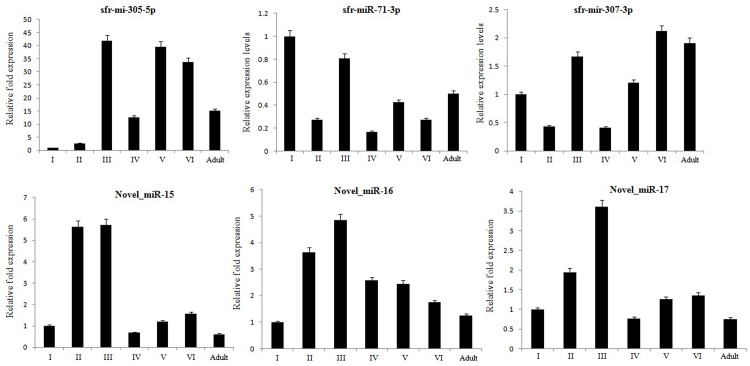
Expression levels of the selected miRNAs in developmental stages of *S. litura*. The absolute expression levels of the selected miRNAs with respect to U6snRNA (endogenous control) were analyzed by TaqMan miRNA assays using the total RNA isolated from each stage of the insect, *S. litura*. The bar graphs represent the expression level of each miRNA at a particular stage in comparison to the expression level in 1^st^ instar larvae. The statistical significance of the qRT analysis was determined by P value < 0.05 while the error bars represent the SD.

As observed from our results (Fig. 5), sfr-mir-305-5p is highly expressed as compared to the other known and novel miRs. For each miR, the expression levels presented in later stages were in comparison with the expression level recorded in 1^st^ instar. All the miRs, except sfr-mir-71-3p, increased in their expression levels during 3^rd^ instar. In addition to sfr-mir-305-5p, sfr-mir-307-3p displayed an increase of 2 fold and more in its expression levels in 6^th^ instar larvae. Though, the three novel miRs, Novel_miR-15, Novel_miR-16 and Novel_miR-17 showed an increase of an approximate 2 folds and more in their expression levels during 2^nd^ instar, only Novel_miR-15 showed more than 5 fold increase in its expression level which is even higher than that of sfr-mir-305-5p. Interestingly, expression levels of sfr-mir-71-3p are significantly lower during all stages except during the 3^rd^ instar. Also the novel miRs, miR-15, miR-16, mir-17 showed similar pattern in expression levels, considering an elevated expression from 1^st^ to 3^rd^ instar while there is a gradual decrease or moderate change during the subsequent stages of development. These results indicate that sfr-mir-305-5p could be a possible regulator for tissue development. Also, the different patterns of the other miRs indicate their diverse pathways related to functions during development.

### Tissue distribution of miRNAs in *S. litura* larvae

Tissue specific expression of a transcript and changes in its levels in different tissues has been a well studied phenomenon of small RNA mediated regulation of gene expression in many organisms. Hence, we studied the expression levels of 6 miRs, both known and novel, in different tissues of 6^th^ instar larvae, specifically in haemolyph, whole gut, body tissue (body of the larvae removing whole gut and fat body) and the fat body separately.

**Fig 6:**
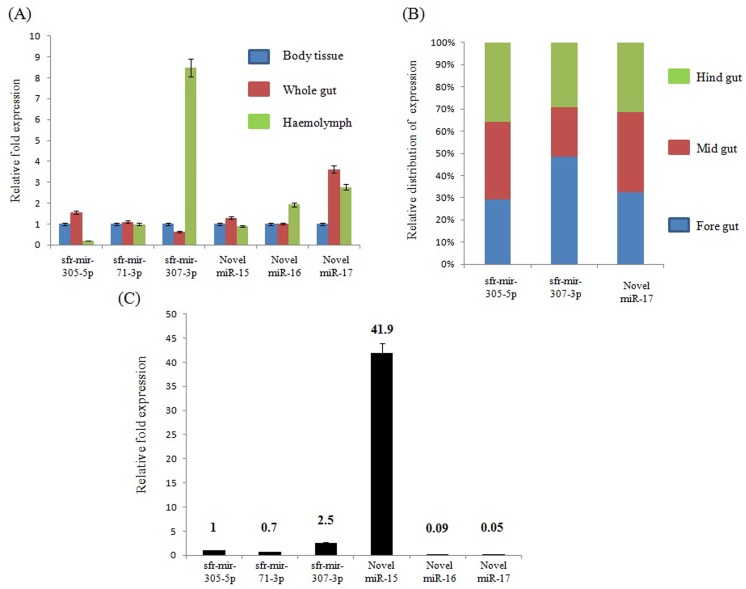
Tissue distribution of selected miRNAs in *S. litura*. The absolute expression levels of the selected miRNAs with respect to U6snRNA (endogenous control) were analyzed by TaqMan miRNA assays using total RNA isolated from respective tissue parts of the larvae. The bar graphs (A, C) represent the expression level of each miRNA in a particular tissue part but in comparison to that of miR-305-5p. (B) The bar graph represents the relative distribution of expression levels of each miRNA in three segments of the whole gut, i.e., fore gut, mid gut and hind gut. The statistical significance of the qRT analysis was determined by P value < 0.05 while the error bars represent the SD.

As shown in the Fig. 6A, sfr-mir-307-3p is highly expressed in haemolymph and expressed less in whole gut while there is a moderate to significant increase in the expression levels of sfr-mir-305-5p and Novel_miR-17 in the whole gut respectively. In addition, we performed the expression check for sfr-mir-305-5p, sfr-mir-307-3p and Novel_miR-17 in three segments of the whole gut, i.e., fore gut, mid gut and hind gut. As shown in the Fig. 6B, there is no significant change in distribution of expression in case of sfr-mir-305-5p and Novel_miR-17 while there is a moderate change in distribution of expression in case of sfr-mir-307-3p. Particularly, sfr-mir-307-3p levels are less in midgut while the expression is mostly constrained to the foregut followed by hindgut. These results implicate that, each miR is slated to regulate a wide variety of transcripts in different tissues of the larvae to maintain the equilibrium in metabolic regulation.

Further, we analysed the expression levels of all 6 miRs in fat body tissue of 6^th^ instar larve. Fat body is a source of energy generation and the levels of metabolic activity are highly complex, consisting of many factors whose levels are tightly regulated. In view of this, we investigated the expression levels of the six miRs separately from the other tissue types. As shown in Fig. 6C, Novel_miR-15 is highly expressed when compared to the other miRs, and sfr-mir-307-3p is approximately 2 fold higher compared to sfr-mir-305-5p. Interestingly, the expression levels of miR-16 and mir-17 are at negligible levels while sfr-mir-71-3p showed a moderate change in its expression when compared to sfr-mir-305-5p. These results clearly indicate that Novel_miR-15 is the predominantly expressed in fat body implicating its role in fat body metabolism of the armyworm while the absence of Novel_miR-16 and Novel_miR-17 is suggestive about the tissue specific expression of miRs and is in agreement with previous findings [5].

### Target prediction of miRNAs

**Fig 7:**
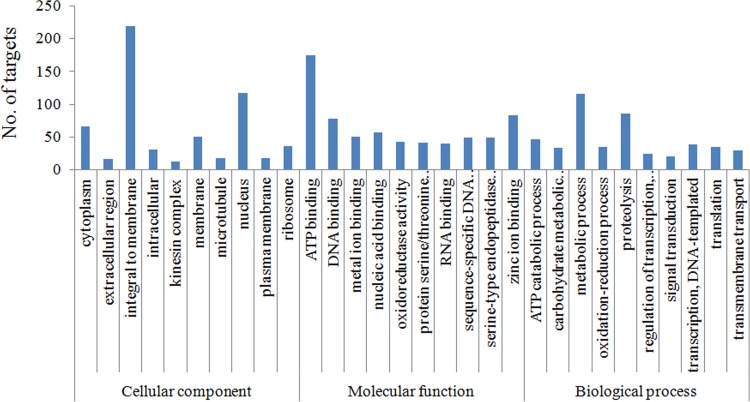
Gene ontology classification of the target genes for the selected miRNAs. Gene ontology (GO) term was assigned to each target gene based on the annotation and were summarized into three main GO categories (biological process, cellular component, molecular function) while only the top 10 sub categories are presented.

miRs regulate protein expression of genes based on the level of complementarity between miRNA seed sequences and the target mRNA [45, 46, 47]. In animals, the seed sequences of miRNA bind to complementary sites on 3’ UTR of its targeted gene. Recent findings have also shown multiple binding sites of mRNA-miRNA interaction, namely in the 5’ untranslated region, 3’ untranslated region and the coding region [48, 49]. Here, we tried to identify the possible target genes for the selected miRs. We used the predicted gene information from the draft genome assembly of Sf21 cells to extract both coding region as well the UTR information for each gene. We performed the analysis for 12 miRs using RNA Hybrid software [50] with MFE cut off of -25Kcal/mol for mi:mRNA binding and identified 809 potential target genes with gene ontology (GO) terms. GO annotation and KEGG pathway analysis were performed to identify functional modules regulated by these miRs. The GO annotation enrichment results showed that component integral to membrane; nucleotide binding function and metabolic process are the most significantly enriched GO terms (Fig. 7). The KEGG pathway analysis revealed 123 pathways that were associated with the potential miR targets. Ribosome, oxidative phosphorylation, RNA transport, protein processing in Endoplasmic Reticulum (ER) and purine metabolism ranked in top most enriched pathways (Table 1). To fully understand the mi:mRNA relation in defining the insect physiology, further validation of the potential target genes both in the cell line as well in different tissue parts of the insect is warranted while the information can be explored to find candidates for insecticidal activity.

**Table 1:** The top KEGG pathways of potential target genes for the validated miRNAs.

ko_ID	Pathway_Name	Number_of_KO
ko03010	Ribosome	48
ko00190	Oxidative phosphorylation	46
ko03013	RNA transport	45
ko04141	Protein processing in endoplasmic reticulum	44
ko00230	Purine metabolism	43
ko03040	Spliceosome	42
ko04120	Ubiquitin mediated proteolysis	41
ko03015	mRNA surveillance pathway	37
ko01200	Carbon metabolism	30
ko03018	RNA degradation	27
ko00240	Pyrimidine metabolism	25
ko04142	Lysosome	24
ko04146	Peroxisome	23
ko04144	Endocytosis	21
ko04310	Wnt signaling pathway	20
ko03008	Ribosome biogenesis in eukaryotes	19
ko03022	Basal transcription factors	19
ko00280	Valine, leucine and isoleucine degradation	18
ko04350	TGF-beta signaling pathway	17
ko00480	Glutathione metabolism	15

## Conclusion

The present study provides an evidence for the expression of miRs in Sf21 cells in significant proportions which are part of small RNA network. We investigated the miR population in *S. frugipdera* cells and studied their expression pattern in *S. litura* lavae. Our analysis revealed that, miRs from *S. frugiperda* were more homologous to *B. mori* miRs when compared to the other insects; *D. melanogaster, T. castenum, A. aegypti* and *A. gambie*. Even the seed region and precursor sequences of the selected miRs were well conserved with *B. mori*, making miRs, a hallmark of evolutionary conservation. Moreover, some of the novel miRs formed clusters on different scaffolds, implicating the necessity of their co-expression. Further, expression levels of 12 miRs (both known and novel) were confirmed by northern analysis and the expression pattern of 6 miRs during developmental stages of the insect as well different tissue parts of the 6^th^ instar larvae added to the notion that, the expression levels of miRs were regulated as per the stage and the type of tissue. In addition, *in silico* analysis of potential targets for the selected miRs revealed that, they were associated with several KEGG pathways. Identification of these potential targets would make basis for their validation to continue with further studies in understanding the biology of *S. frugiperda*.

### Data accession

The genome data of Sf 21 cell lines used in the present study is available at NCBI WGS under the accession number: JQCY00000000.

## Supporting Information

S1 Table Information on all the known and novel miRNAs identified in the present study from Sf21 cells. (XLS)

S2 Table Information on the abundance of novel miRNAs present in the clusters identified in the present study on different genome scaffolds of Sf21 cells. (XLS)
